# Soft Tissue Reconstruction with Free Gingival Graft Technique following Excision of a Fibroma

**DOI:** 10.1155/2015/248363

**Published:** 2015-08-18

**Authors:** Nurcan Tezci, Suleyman Emre Meseli, Burcu Karaduman, Serap Dogan, Sabri Hasan Meric

**Affiliations:** ^1^Department of Periodontology, Faculty of Dentistry, Istanbul Aydin University, 34295 Istanbul, Turkey; ^2^Department of Pathology, Faculty of Medicine, Istanbul University, 34093 Istanbul, Turkey

## Abstract

*Background*. Oral fibromas are benign, asymptomatic, smooth surfaced, firm structured tumoral lesions that originate from gingival connective tissue or periodontal ligament. Histologically, they are nodular masses characterized by a dense connective tissue, surrounded by stratified squamous epithelium. *Case Report*. This case report includes the clinical, radiographical, and histological findings and periodontal treatment of a 38-year-old female patient having painless swelling on the gingiva. Intraoral examination revealed a fibrotic, sessile, smooth surfaced gingival overgrowth interdentally between the teeth #13 and #14. Radiographical findings were normal. Initial periodontal treatment (IPT) was applied including oral hygiene instructions, scaling, and root planing. Following IPT, the lesion (0.7 × 0.6 × 0.4 cm) was excised and examined histopathologically. Subsequently, flap operation was performed to have an access to alveolar bone. Surgical site was reconstructed with free gingival graft obtained from hard palate. Hematoxylin-eosin stained sections revealed a nodular mass composed by dense collagen fibers in lamina propria covered by a stratified squamous epithelium, which were consistent with fibroma. Gingival healing was uneventful and without any recurrence during the 12-month follow-up. *Conclusions*. In order to achieve optimal functional and aesthetical outcomes, free gingival graft can be used for the reconstruction of the wound site after the excision of the fibroma.

## 1. Introduction

Reactive soft tissue lesions are commonly seen in routine dental practice. Oral fibroma (OF) is a benign tumoral lesion originating from gingival connective tissue or periodontal ligament. The aetiology of various reactive hyperplasias like OF is chronic, irritating factors such as calculus, overhanging restoration margins, self-injury, food impaction, extended flanges of dentures, or broken teeth [1]. OFs have been diagnosed in 4% of the oral soft tissue lesions [2].

OF is generally asymptomatic and clinically has a smooth surface. OFs are generally pedunculated, slow-growing, and spherical enlargements tending to be firm and nodular but also soft and vascular [3]. OFs usually have no radiographic features. However, in some cases, cup-shaped radiolucency occurs in the underlying bone [4].

OFs are composed of bundles of well-formed collagen fibers with a scattering of fibrocytes and a variable vascularity. Histologically, they are nodular masses characterized by the presence of a dense connective tissue surrounded by stratified squamous epithelium [5].

OFs are treated by excisional biopsy to eliminate the whole lesion from the basement in order to prevent recurrence. In some cases, aggressive excision extending to the bone must be performed to eliminate the possibility of recurrence [6].

In this case report, it is aimed to present the clinical, radiographical, and histological findings and periodontal treatment approach of an OF on the maxillary buccal gingiva.

## 2. Case Report

This case report includes the clinical, radiographical, and histological findings and periodontal treatment of a 38-year-old female patient who consulted the Department of Periodontology, Faculty of Dentistry, Istanbul Aydin University, with a complaint of painless swelling on the gingiva. She was systemically healthy and had not taken any medicine/over-the-counter remedies that could have influenced the gingival overgrowth. The patient had no child and she was not pregnant at the time of clinical examination.

**Figure 1:**
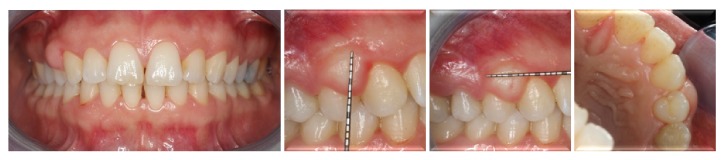
Clinical appearance of the whole mouth and lesion.

**Figure 2:**
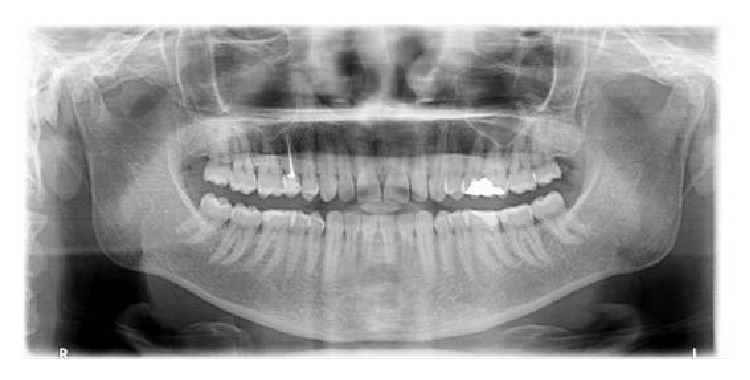
Radiographical image of patient.

Intraoral examination revealed a fibrotic, sessile, and smooth surfaced gingival overgrowth interdentally between the teeth #13 and #14 (Figure 1). Moreover, slight plaque accumulation, mild gingival inflammation, and edema were observed. Radiographical findings were normal (Figure 2). Plaque-associated gingivitis was diagnosed based on the clinical and radiographical examinations. Full-mouth clinical periodontal parameters were as follows: mean plaque index 1.09 ± 0.69, gingival index 1.11 ± 0.66, and probing depth 2.92 ± 1.09. The teeth #13 and #14 had no mobility and no response to horizontal and vertical percussion tests and were vital based on the results of electrical pulp test. The patient signed a detailed written consent about treatment plan, postoperative healing, and complications.

Initial periodontal treatment (IPT) consisting of oral hygiene instructions, scaling, and root planing was applied. However, IPT had slight effect on the recovery of the lesion. The clinical and radiographical findings revealed that the preliminary diagnosis of the lesion was found out to be fibroma.

**Figure 3:**
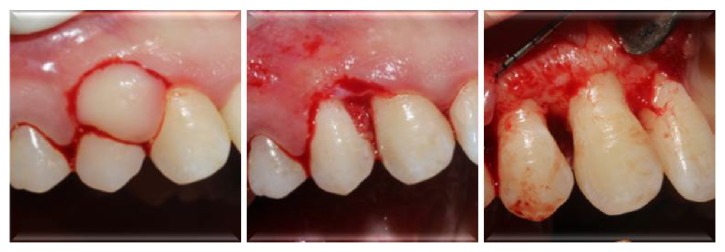
Excision of the lesion and flap operation of the site.

**Figure 4:**
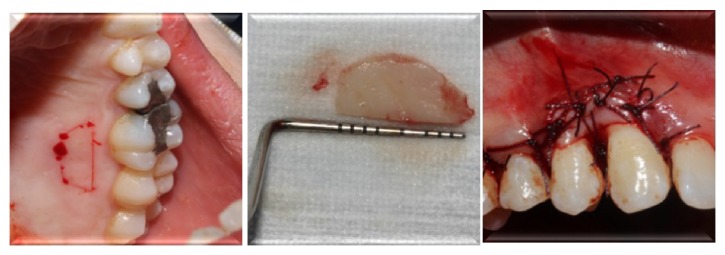
Reconstruction of surgical site with free gingival graft.

Following IPT, the lesion (0.7 × 0.6 × 0.4 cm) was excised and examined histopathologically. Subsequently, flap operation was performed to gain access to alveolar bone (Figure 3). Sulcular incisions were made buccally and palatally without any vertical incision. Buccal and palatal mucoperiostal flaps were reflected and all granulation tissues were removed. Resective bone surgery was performed in order to eliminate the lesion without leaving any mineralized material which may cause recurrence. Surgical site was reconstructed with free gingival graft obtained from hard palate to ensure primary closure of the flaps and prevention of gingival recession (Figure 4).

Flaps were sutured with 4/0 silk suture material (Dogsan İpek 4/0, 16 mm, 3/8, cutting suture, Trabzon, Turkey). Following surgical operation, amoxicillin/clavulanic acid 2*∗*1 for 5 days (Augmentin-BID 1000 mg 10 tablets, GlaxoSmithKline, London, UK), naproxen sodium 2*∗*1 for 5 days (Apranax Fort 550 mg 10 film tablets, Abdi İbrahim, İstanbul, Turkey) and chlorhexidine gluconate oral rinse 2*∗*1 for 5 days (Andorex 120 mL Delta Vital, Istanbul, Turkey) were prescribed to prevent any possible postoperative complication [7]. Clinical healing was observed without any postoperative complications such as pain, hemorrhage, and swelling.

**Figure 5:**
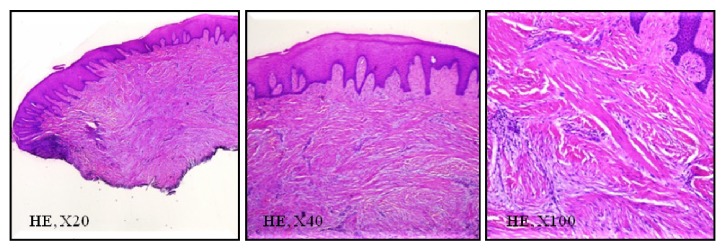
Histopathological sections of the excised lesion with different magnification.

Removed tissue sample was fixated in 10% formaldehyde solution, routinely processed, sectioned, and stained with hematoxylin-eosin. Stained sections revealed a nodular mass composed by dense collagen fibers in lamina propria covered by a stratified squamous epithelium, which were consistent with OF (Figure 5).

**Figure 6:**
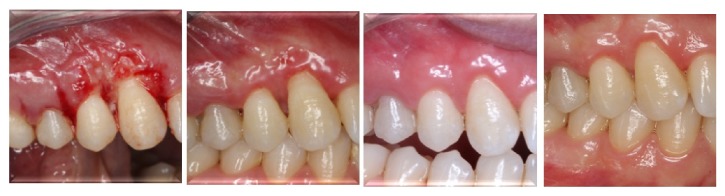
Postoperative 1st week's and 1st, 6th, and 12th months' clinical appearance of the surgical site, respectively.

Gingival healing was uneventful and without any recurrence during the 12-month follow-up period (Figure 6).

## 3. Discussion

OF is a benign lesion that occurs against the local and chronic trauma-affected area. Most of the lesions are seen in the third decade and more prevalent in females than in men. A higher prevalence in female may indicate the role of hormones as predisposing factors in the development of this lesion [8]. Zarei et al. studied [8] 172 cases of reactive hyperplasias and found the mean age of the patients to be 37 years. Maxillary gingivae is the most common site for fibrous lesion development among the periodontal tissues and these lesions are not found in the palatal area [8]. In our case, the patient was 38 years old and the localization of the lesion was the maxillary buccal gingiva, as mentioned in the reported literature [8, 9]. Differential diagnosis of the OF is histologically made with pyogenic granuloma, peripheral giant cell granuloma, and peripheral ossifying fibroma [9].

Early detection and treatment of reactive lesions can reduce dentoalveolar complications such as malignant transformation [9]. Misdiagnosis of a malignant transformation of a reactive lesion may lead to a wrong or deficient treatment plan designed to eliminate these type of lesions. Therefore, identifying the frequency and distribution of these lesions is beneficial for establishing a diagnosis and a proper treatment plan in practice. Generally, the treatment of OFs is performed by the excision of the lesion. In some cases, flaps must be reflected to gain access to alveolar bone and prevent recurrence of lesions derived from mineralized tissues [10]. If any defect remains at the surgical area, soft tissue reconstruction may be preferred to minimize the loss of keratinized tissue width and to achieve better aesthetical results [6]. Walters et al. [6] treated 3 peripheral ossifying fibroma lesions, one of which had two conservative excisions before, where recurrence was observed. In order to prevent this, they made incisions extending into the adjacent bone tissue. After the excision of these 3 peripheral ossifying fibroma lesions, gingival defects of each lesion were treated with different plastic surgical procedures: laterally positioned flap, subepithelial connective tissue graft combined with a coronally advanced flap, and coronally advanced flap. The lesions did not recur during the long-term follow-up [6].

Free gingival graft operation is an excellent and predictable surgical procedure to protect adequate keratinized tissue width [11]. In our case, following the excision of the lesion, free gingival graft was placed over the wound site to ensure the primary closure of the flaps. Patel et al. [9] used the same approach to treat a peripheral odontogenic fibroma case and obtained a favorable outcome where lesion recurrence was observed. Using free gingival graft is a preferable technique in order to reconstruct the wound site after excision of a fibroma. Beyond surgical approaches, optimal plaque control during the postoperative period is crucial to obtain favorable outcomes. Hence, the patient must be motivated by the clinician to achieve better results.

With this treatment approach in our case, there was no recurrence after 12 months of follow-up and the functional and aesthetical outcomes were satisfactory for the patient and the clinicians. It is emphasized in this case report that excision of oral soft tissue lesions may result in defects in the aesthetical regions of the oral cavity. So, the clinicians must consider this clinical problem while deciding optimal treatment plan.
